# The VersaJet for Breast-Reduction Surgery: Operator Beware

**Published:** 2012-02-06

**Authors:** Karan Chopra, Matthew K. Folstein, Sheri Slezak, Ronald Silverman, Devinder Singh, Brian R. Gastman

**Affiliations:** ^a^Department of Surgery, University of Maryland School of Medicine, Baltimore; ^b^Department of Surgery, University of Maryland Medical Center, Baltimore; ^c^Division of Plastic Surgery, University of Maryland Medical Center, Baltimore; ^d^Department of Plastic Surgery, Dermatology and Plastic Surgery Institute, Dual appointments in: Taussig Cancer Center, Lerner Research Institute, Department of Immunology, Head and Neck Institute, Cleveland, Ohio.

## Abstract

**Background:** Modern techniques for breast-reduction surgery depend on large surface de-epithelialization. The current gold standard technique involves the use of a scalpel for sharp debridement and is a time-consuming process that is labor-intensive and often requires an assistant to stabilize the breast. Surgeons who perform breast-reduction surgery continue to search for instruments and innovations that may reduce the time and effort required for successful de-epithelialization. One such innovation is the use of the VersaJet Hydrosurgery system. The purpose of this article is to highlight an important complication that may result from the use of this device. **Methods:** The authors describe their experience with the VersaJet Hydrosurgery System in reduction mammoplasty of 28 breasts. **Results:** The authors experienced several complications characterized by the formation of epithelial inclusion cysts requiring reoperation. **Conclusion:** The VersaJet Hydrosurgery system may lead to quicker speeds of de-epithelialization as compared with traditional methods but poses a risk of epidermal cyst formation.

Large surface de-epithelialization is a labor-intensive component of breast-reduction surgery. Although many surgeons successfully use the traditional scalpel technique, many others have applied novel strategies and equipment to hasten this process with mixed results. For instance, some surgeons report an increased speed of de-epithelialization when using the dermatome. However, this method is prone to inconsistent results related to tissue skipping.^1^ Another proposed technique for de-epithelialization is the VersaJet Hydrosurgery system.^1^^,^^2^ In our experience, the VersaJet led to the development of epithelial inclusion cysts that required reoperation for resolution. Thus, the VersaJet should be used with caution when attempting to de-epithelialize the breast for reduction mammoplasty.

Our division experienced favorable outcomes while using the VersaJet Hydrosurgery System for tissue debridement in the setting of pressure ulcers and burn wounds. This instrument allowed expeditious debridement of large areas of granulation tissue in ulcerative wounds to prepare for skin grafting. Our results in this context were positive and consistent with the successful outcomes reported in the literature.^3^^,^^4^ Similarly, there is at least 1 report showing that the VersaJet allows for selective removal of necrotic epithelium, while preserving vital dermal planes.^5^ A novel use in the literature was for de-epithelialization of the breast for breast-reduction surgery.^2^ Lonergan and Moquin^2^ reported successful outcomes in 20 patients with no complications over a 6-week to 8-month follow-up period. It was in light of our own positive experiences with the VersaJet and the positive outcomes in the literature that we attempted to apply this device to breast-reduction operations.

The VersaJet technique allowed for what appeared to be uniform de-epithelialization in a single plane of tissue. Unlike with the use of a blade, there was no dulling; thus, the operation proceeded uninterrupted and the de-epithelialization phase lasted approximately 10 minutes. Despite these benefits, in our sample size of 14 patients, 9 patients experienced no negative outcomes while 5 patients experienced serious complications. In contrast to the results of the Lonergan and Moquin study of 20 patients, which revealed no complications,^2^ we encountered 5 patients who suffered local wound complications attributed to epidermal cyst formation that were midline and around the nipple-areolar complex. These patients presented at 12 to 16 weeks postoperatively, well beyond the follow-up period stated in the Lonergan study. In at least 4 cases, these cysts were extensive enough to require reexploration and resection for definitive treatment. Upon reexploration of these cysts, we observed multiple epithelial tracts in the area of the cyst. As a result, we must reconsider the applicability of this novel technique in obtaining replicable, sustainable, and positive long-term results. At the time, we performed 1 to 2 passes with the VersaJet, which removed any obvious epithelium. It is possible that breasts that are de-epithelialized using this technology are susceptible to epidermal cyst complications because the VersaJet does not remove the epidermal layer completely or it sprays the epidermal cells to adjacent tissue. At a minimum, the operator is urged to perform 3 to 4 passes with the VersaJet and then carefully inspect the breast for any remaining epidermis. Until the use of this tool is standardized in terms of depth of debridement, the VersaJet for breast-reduction surgery should be used with great caution.

A review of the literature yields only a few articles discussing the depth required to properly de-epithelialize. Older reports of dermal pedicle biopsies indicate that standard “hand and knife” techniques remove the epidermis and a variable amount of dermis.^6^^-^8 A study published in 1969 evaluated the fate of buried free dermal fat grafts. This report documented that residual skin appendages such as sweat glands and hair follicles gradually atrophy and are replaced by fibrous tissue.^9^ What was clear from pathologic specimens from buried dermal pedicles was that the pilosebaceous apparatus (but not the deepest sweat glands) was removed with the “de-epithelialization.” Ultimately, all authors agreed that the term *de-epithelialization* was a suboptimal descriptor for VersaJet Hydrodissection of the breast pedicle.

The VersaJet Hydrodissector, while offering increased speeds of de-epithelialization over traditional methods, presents a risk of epidermal cyst formation that cannot be ignored. Although we are enthusiastic about the potential uses for this machine, we feel the need to highlight our complications to the plastic surgery community. Further studies will be required to investigate the histological changes in the breasts with epidermal cyst formation, and an investigation to determine the depth of de-epithelialization afforded by the VersaJet system. Perhaps future devices may use the fundamentals behind the hydrodissector to create a safe de-epithelialization device for breast-reduction surgery. Breast surgeons performing breast-reduction surgical procedures should be cautious before implementing this technology.
